# Developing an integrated brain resource framework for translational neuroscience in Korea Brain Bank

**DOI:** 10.3389/fneur.2026.1771354

**Published:** 2026-04-10

**Authors:** Heon Seok, Taekwon Son, Yeon Jin Ryu, Hee Jeong Yoon, Sung-Uk Kim, Eum-Ji Kim, Greg T. Sutherland, Ja Wook Koo, Kipom Kim, Pann-Ghill Suh

**Affiliations:** 1 Korea Brain Bank, Korea Brain Research Institute, Daegu, Republic of Korea; 2 Emotion, Cognition and Behavior Research Group, Korea Brain Research Institute, Daegu, Republic of Korea; 3 New South Wales Brain Tissue Research Centre, School of Medical Sciences and Charles Perkins Centre, Faculty of Medicine and Health, The University of Sydney, Camperdown, VIC, Australia; 4 Korea Brain Research Institute, Daegu, Republic of Korea

**Keywords:** K-brain net, Korea Brain Bank, multi-omics, sociocultural barriers, translational neuroscience

## Abstract

The Korea Brain Research Institute (KBRI) and its core facility, the Korea Brain Bank (KBB), are establishing a national framework that integrates human brain banking with digital and multi-omics resources to strengthen translational neuroscience in Korea. Operating under the Brain Research Promotion Act (BRPA) and supported by the Ministry of Science and ICT (MSIT), the KBB manages the designated Korean Brain Bank Network (KBBN), a national system that provides centrally coordinated nationwide coverage through eight network hospitals. The network is supported by the KBRI-developed Brain Resource Archive Management System (BRAMS) for integrated management of human brain resources. KBBN enhances interoperability, standardization, and data accessibility across the network. These efforts have led to measurable improvements in data completeness, resource utilization efficiency, and research connectivity. Since its establishment, KBBN has prioritized the collection of brain resources from patients with neurodegenerative disorders, enabling neuropathological assessment and digital dataset construction for integrated analysis. However, securing brain resources for psychiatric disorders remains limited due to sociocultural barriers to donation in Korea. To address this, KBB is expanding international collaboration to develop specialized psychiatric cohorts for joint multi-omics studies. Future strategies focus on increasing functional resource value through single-cell and spatial omics integration, linking postmortem-derived iPSC and organoid models, and establishing an AI-assisted federated data governance system that integrates imaging, genomic, and clinical data within a Brain Atlas Hub. Through the convergence of cellular innovation, digital governance, and global cooperation, KBB seeks to advance responsible and sustainable brain resource management in Korea.

## Introduction

The 21st century presents an unprecedented biomedical challenge: accelerating population aging and the rising burden of neurodegenerative disease. Korea, one of the fastest-aging societies, is projected to become a super-aged society by 2025, with more than 20% of the population over 65 years old (1). Dementia prevalence in Korea surpassed 1 million cases in 2024 and is projected to reach 1.4 million by 2030 (2). The annual socioeconomic burden of neurological diseases now exceeds KRW 30 trillion (approximately USD 22.2 billion) (3), underscoring an urgent national public health priority (4, 5).

Progress in disease-modifying therapeutics, however, has been limited by the difficulty of translating findings from experimental models to human brain pathology. Human brain biobanking addresses this bottleneck by enabling direct investigation of molecular pathology, cell-type–specific changes, and circuit-level vulnerability in well-characterized postmortem tissue, while supporting biomarker validation and target discovery in a clinically anchored context (6–8). As a result, modern brain banks are increasingly viewed not only as repositories, but as integrated research infrastructures linking biospecimens with standardized neuropathology, imaging, and multi-omics data.

Within this landscape, Korea established the Korea Brain Bank (KBB) and the national Korean Brain Bank Network (KBBN) under the Brain Research Promotion Act (BRPA) to strengthen national research sovereignty and provide standardized, accessible brain resources. This perspective summarizes the decade-long evolution of the KBB (2014–2025), highlighting its institutional development, resource and cohort strategies, digital integration through Brain Resource Archive Management System (BRAMS), and future directions for sustainable growth and global collaboration.

## The development of the Korea Brain Bank: evolution into a national research infrastructure

Established in 2014 under the Korea Brain Research Institute (KBRI), KBB has developed into a national infrastructure supporting brain disease research. Through successive phases of infrastructure building, network expansion, and digital modernization, the KBB has progressed from an initial focus on tissue acquisition and preservation to a researcher-oriented platform enabling access, distribution, and utilization of brain resources. In 2023, the Ministry of Science and ICT (MSIT) designated the KBB as Korea’s first national brain bank, reinforcing its role within the national brain research ecosystem.

In 2014, the KBB was approved as a Human Bioresource Bank, establishing core workflows for the collection and preservation of brain tissue. Through collaborations with major hospitals, it initiated a brain donation program to secure essential materials for neuroscience research. Between 2015 and 2016, the KBBN was launched through the pilot KBBN Support Project, connecting regional hospitals and forming a nationwide framework for donation and distribution. The Phase I, KBBN Operational Support Program (2014–2020) further strengthened nationwide collection capacity, enabling the KBB to function as a national research hub beyond simple storage. Following its 2017 designation as an Authorized Biological Research Resource Repository, the KBB accelerated its digital transformation. In 2018, it launched the KBBN Portal and the Brain Resource Management System (BRAMS 1.0), enabling online discovery and access to brain resources. Neuropathology Research Committee and advisory activities initiated in 2019 strengthened quality assurance and translational research support.

The Phase II Support Program (2021–2025) strengthened governance and stabilized core operations across the network by formalizing standardized workflows, quality management, and multi-site coordination procedures. KBB expanded both operational scale and digital capability. In 2021, the Brain Cluster Development Project further enhanced systems for resource collection and utilization. Building on these efforts, KBB launched the K-BrainNet Portal and BRAMS 2.0 in 2024, introducing more automated and functions to share and access standardized resource information and linked datasets more securely and efficiently, improving workflow efficiency. Together, these upgrades established a more researcher-friendly system for discovering and accessing available resources and linked data.

As of December 2025, KBB has secured 3,362 donor registrations and 379 postmortem brain donations and has collected 2,948 pre-mortem biospecimen cases (CSF, blood, urine). In parallel, the KBB is building a genomic dataset comprising 368 cases, including whole-exome sequencing (WES) for epilepsy and developmental disorders, as well as GWAS and large-scale targeted sequencing for Alzheimer’s disease. The continued expansion of both physical biospecimens and molecular data represents an important step toward systematic national governance of brain research resources.

In parallel, the KBRI has expanded its data infrastructure to support data-driven neuroscience. This effort began in 2021 with Phase I of the Brain Research Data Station and was reinforced by the establishment of the KBRI Brain Tech Center in 2023. Phase II, launched in 2025, focuses on enhancing data accessibility and cross-platform integration to advance a comprehensive digital biobanking ecosystem.

**Figure 1:**
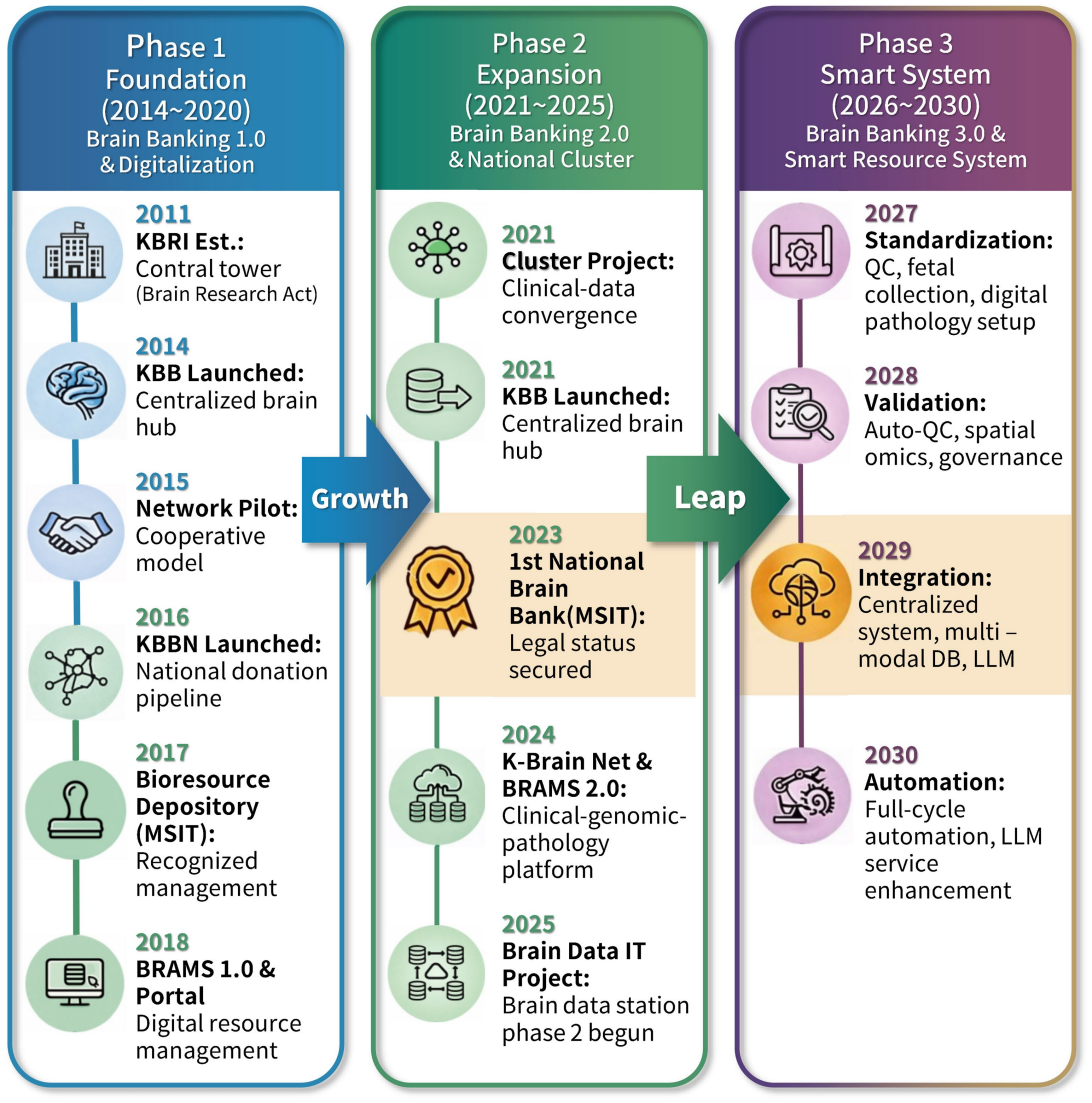
Korea Brain Bank roadmap (2011–2030): evolution from centralized banking to an integrated smart platform.

Overall, KBB’s development over the past decade reflects a shift from foundational establishment to operational and digital maturation. By integrating physical biobanking with digital infrastructure, the KBB aims to serve as a reliable platform for Korea’s neuroscience community. Looking ahead, the initiative will continue to advance toward a “Brain Banking 3.0” framework, with an emphasis on sustainability, interoperability, and responsible innovation (Figure 1).

## Human brain resource acquisition and pathological profiling in the Korea Brain Bank Network (KBBN)

In Korea, the legal and sociocultural environment has traditionally constrained the acquisition of postmortem human tissues for research. Under the Organ Transplantation Act, all organ and body donations—including those from brain-dead individuals—require explicit consent from the donor or their family, in contrast to the presumed-consent models used in several European countries such as Spain, France, Austria, Belgium, and the United Kingdom. In these countries, individuals are considered donors by default unless they opt out, a system that has substantially increased donation rates (9, 10).

By contrast, Korea maintains a family decision-based system in which family awareness, emotional readiness, and understanding of brain death are critical determinants of donation outcomes (11). Despite the establishment of the Korea Organ Donation Agency (KODA) and the national transplant registry (KOTRY), Korea’s deceased organ donation rate remains 8.7 donors per million population (pmp) as of 2018—well below the OECD average of 20–30 pmp (12). This low rate is partly influenced by Confucian values emphasizing bodily integrity and filial piety, which often discourage postmortem interventions (13). National data further show that only 28.9% of medically suitable brain-dead donors proceed to actual donations, with family refusal being the major barrier (14).

In addition to these structural barriers, misunderstanding about brain donation for research may further reduce consent. In an Australian community survey, 50% of respondents who were registered organ donors believed that organ donor registration automatically included brain donation for research, and 55% were not aware that brain donation was possible, highlighting substantial public misconceptions (15). This suggests the need to clearly distinguish brain donation for research from organ donation for transplantation, with separate consent and communication pathways.

Despite these institutional, cultural, and public-facing constraints, the KBBN has made steady progress in establishing a sustainable national brain donation and research infrastructure. Founded in 2015 with only five donations, the expansion of the network—through continuous public outreach, ethical standardization, and hospital partnerships—to 379 postmortem brains by December 2025, reflects a consistent annual growth of 15–30%. The collection encompasses both adult and fetal brain tissues: over 80% of adult donors are aged 60 years or older, providing essential material for studies on Alzheimer’s and Parkinson’s disease, while 26.6% (101 cases) consist of fetal brains (16–25 gestational weeks) supporting research on brain development and congenital disorders.

The associated clinical data are variably complete—approximately 80% for basic demographics, 50–60% for disease history, and lower rates for family medical history (~20%). Neuroimaging data are available for only ~10–15% of cases, and both obstetric information and neurological examination data remain below 10%, underscoring the need for continued improvements in donor registration and data-linkage systems. In contrast, the pathological dataset is highly consistent and standardized, with systematic neuropathological assessments performed on an ongoing basis for all postmortem cases. Following NIA–AA international criteria, neurodegenerative cases are assessed for 11 key indicators, including amyloid plaque distribution (Thal phase), tau pathology (Braak stage), neuritic plaque density (CERAD score), cerebral amyloid angiopathy (CAA), Lewy body pathology, hippocampal sclerosis, and TDP-43 inclusions, forming the basis for standardized classification and final neuropathological diagnosis. As of 2025, the KBB has achieved near-complete neuropathological diagnostic coverage (~100%) across eight major neurodegenerative diseases, and its histological samples have been digitally scanned to establish a national brain pathology atlas comprising 182 cases and 10,690 image datasets, providing a robust foundation for digital pathology. This robust foundation of well-characterized resources has driven a clear growth in utilization. Following an initial establishment phase (2017–2022), distribution volumes have increased substantially since 2023. As of March 2026, 3,259 biospecimens—primarily brain tissue (64.9%) and genomic data (20.0%)—have been distributed to support 133 independent research projects. These resources are utilized mainly for elucidating disease pathogenesis (39.1%), biomarker development (21.1%), and omics analysis (18.1%). Ultimately, these metrics highlight the practical role of our secured resources in supporting and advancing brain research across Korea.

## Global context and strategic vision: tackling the mental health crisis

Korea currently faces a growing mental health burden, marked by a sharp rise in mood disorders and substance use disorders. According to recent national data, the number of patients treated for depression has surpassed 1 million for the first time, reflecting an average annual increase of nearly 7% (3). Drug-related offenses have also reached record highs, challenging the country’s long-standing perception of being “drug-free” (16). In parallel, Korea continues to report the highest suicide rate among OECD nations (17).

Despite this substantial epidemiological burden, elucidating the biological underpinnings of these disorders remains challenging. Global studies have shown that molecular analyses of postmortem brain tissue are important for understanding the neurocircuitry and pathology underlying addiction and affective disorders (18, 19). However, Korea’s research infrastructure in this domain has been constrained by a historical scarcity of well-characterized psychiatric brain resources.

In response, the KBB is broadening its mandate to serve as a specialized platform for psychiatric research. The objective is to strengthen institutional capacity for the systematic acquisition and curation of psychiatric brain tissue, enabling molecular autopsy pipelines and high-resolution genomic analyses. To accelerate this effort, the KBB has deepened partnerships with global institutions, including the Netherlands Brain Bank (NBB), the Stanley Medical Research Institute (SMRI), and the NSW Brain Tissue Resource Centre. Since 2022, these collaborations have progressed from administrative agreements to active joint research initiatives that adopt international best practices. Current activities include joint digital archiving, cross-validation of neuropathological assessments, and metadata harmonization to establish a shared reference framework for psychiatric brain research. Through these efforts, the KBB aligns Korean psychiatric brain resources with international standards guided by the FAIR and MIABIS principles (20, 21). Ultimately, this integration aims not only to standardize resources but also to facilitate discovery-driven research that supports precision psychiatry and suicide prevention efforts.

## Digital integration and future outlook

**Figure 2:**
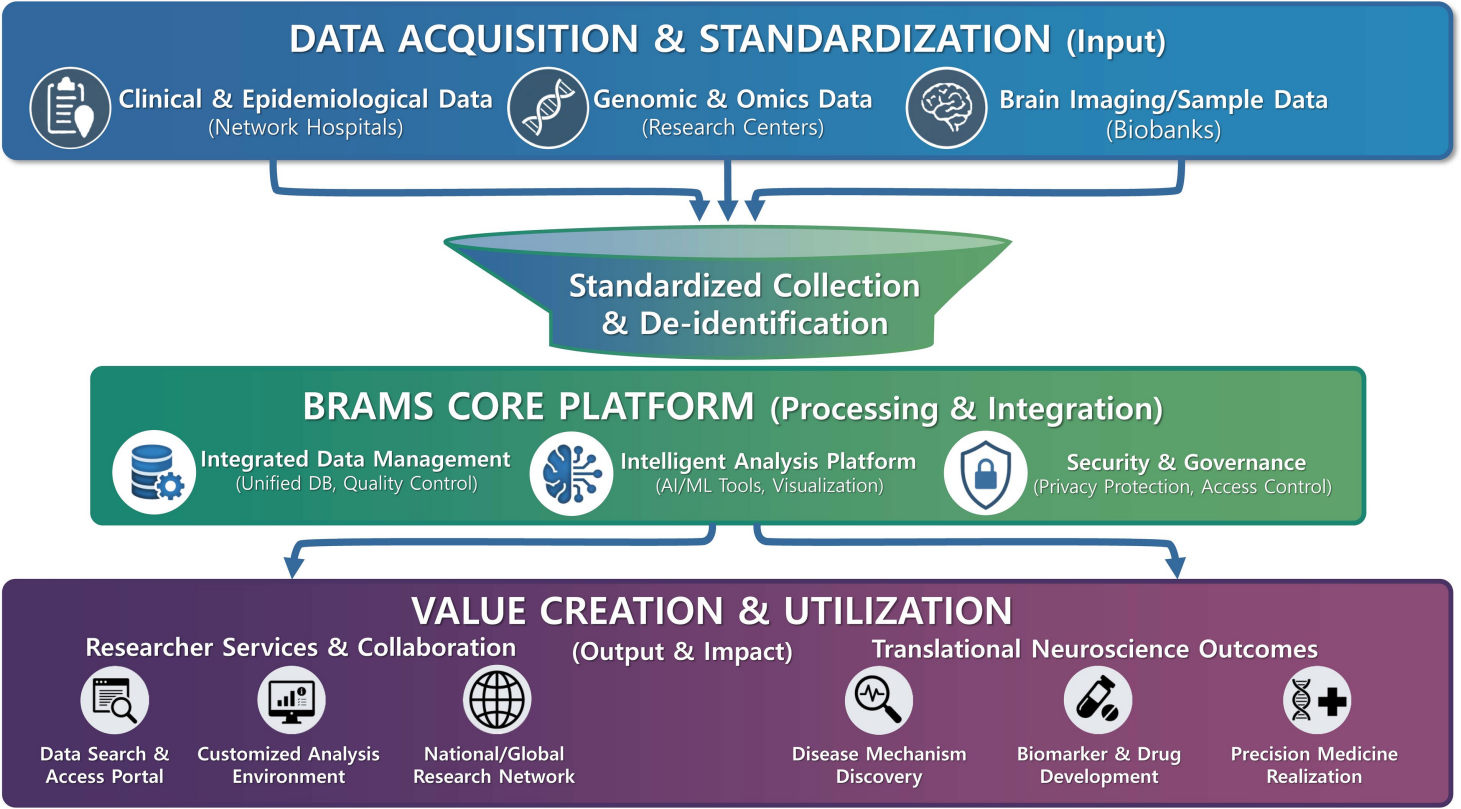
Strategic direction of data operations in BRAMS for translational neuroscience research. This diagram illustrates the overarching flow of data operations within BRAMS. It details the journey from Data Acquisition & Standardization (Input), collecting diverse datasets (clinical, genomic, imaging) through standardized and de-identified protocols. This feeds into the BRAMS Core Platform (Processing & Integration) for unified management and intelligent AI/ML-driven analysis under secure governance. Finally, the flow leads to Value Creation & Utilization (Output & Impact), enabling advanced researcher services and driving tangible translational neuroscience outcomes such as disease mechanism discovery and precision medicine realization.

As neuroscientific data rapidly increases in scale and complexity, the field is shifting from simple archiving toward AI-enabled, multimodal data integration (22). While BRAMS 2.0 currently provides stabilized, standardized digital governance and nationwide resource accessibility, its siloed architecture limits advanced multidimensional analyses. In alignment with Phase III (2026–2030), KBB is planning to transition to the ‘Brain Banking 3.0’ smart resource framework—an evolution intended to address the limitations of isolated datasets. This planned transition will be supported by the development and implementation of BRAMS 3.0, a next-generation federated data architecture. To link phenotypic information with deep molecular signatures, BRAMS 3.0 aims to move beyond traditional silos and provide a unified analytics environment in which histopathology, high-resolution neuroimaging, and multi-omics data can be jointly analyzed (23). This implementation is anchored in the ‘National Bio-Resource Management Digitalization Project (Brain Cluster),’ a government-led initiative. By integrating centralized *de novo* data generation with high-quality returns from the research community, KBB establishes a synergistic framework ensuring both the breadth and completeness of its multi-omics and digital datasets. This dual approach enables the robust, joint analysis of complex neuroscientific resources within the BRAMS 3.0 ecosystem (Figure 2).

Rapid advances in spatial transcriptomics and reconstruction are also driving brain atlases to evolve from static 2D maps toward dynamic digital-twin representations (24, 25). Building on these advances, KBB plans to develop a multi-dimensional Brain Atlas Hub that spatially aligns 3D pathology data with single-cell gene expression maps, enabling molecular pathology to be interpreted within precise anatomical context. In parallel, the KBB addresses a core limitation of traditional biobanking: postmortem tissue reflects a static, end-stage snapshot of disease, limiting the study of functional progression. To help bridge static pathology and dynamic function, the initiative is adopting a “Living Biobank” approach. By generating patient-derived iPSCs and brain organoids from the same donor pool, the KBB can establish a functional validation loop—allowing molecular hypotheses derived from postmortem data to be tested in personalized, living models—thereby strengthening the translational workflow (26).

Finally, as data sovereignty and privacy regulations (e.g., GDPR) tighten, cross-border transfer of sensitive data is becoming increasingly constrained. To address this, KBB is implementing federated learning (FL) protocols that enable international AI models to learn from Korean data while maintaining data locality and privacy protection (27). This framework supports interoperability with global initiatives such as the BRAIN Initiative and EBRAINS, positioning KBB not only as a national repository but as a federated node in a global translational neuroscience network—aligned with FAIR principles and compliant with relevant privacy regulations.

As we work toward this vision, our early operations have highlighted unique considerations. The cultural sensitivities surrounding mental health in Korea necessitate a highly delicate family consent process, requiring tailored approaches for psychiatric tissue acquisition. Furthermore, for the post-mortem brain resources we have secured, achieving true data completeness presents a distinct operational hurdle. Because psychiatric care is heavily decentralized, patients’ pre-mortem clinical records are often dispersed across multiple independent clinics. Therefore, systematically harmonizing these fragmented clinical trajectories into comprehensive datasets remains an essential, ongoing objective for our network.

## Discussion

Over the past decade, the KBB and KBBN have progressed from conventional biorepository operations to a digitally integrated, data-driven infrastructure supporting translational neuroscience. By implementing standardized workflows, interoperable databases, and BRAMS-enabled digital governance, the KBB has strengthened the reproducibility and long-term viability of human brain research in Korea. These developments align with the broader global shift toward federated neurodata ecosystems that connect imaging, neuropathology, and multi-omics modalities, guided by FAIR principles and compliant with GDPR and related privacy regulations (20, 28).

Recent advances in AI-driven data harmonization and living-biobank approaches underscore the need for adaptive biobank infrastructures that can incorporate emerging paradigms—from patient-derived organoids to large-scale foundation models—while preserving ethical oversight, provenance, and interoperability (28, 29). In this context, a Brain Banking 3.0 framework aims to implement an AI-assisted, modular architecture that can accommodate new analytical methods without compromising governance and cross-platform compatibility, including an large language model (LLM)-enabled portal for natural-language search and cross-linking of standardized resource metadata (images, omics, and clinical records), as well as AI-assisted digital pathology workflows for automated quality control, pattern detection, and quantitative readouts from whole-slide images (30, 31). This evolution is expected to support interoperable, cross-site analyses and facilitate international collaboration across neurodegenerative, neuropsychiatric, and other neurological disease domains.

Collectively, the KBB’s adaptive, ethically grounded, and digitally integrated approach provides an implementation-oriented example that may inform ongoing international efforts to advance responsible and collaborative brain science with emerging analytical methods incorporated under robust governance frameworks.

## Data Availability

The original contributions presented in the study are included in the article/supplementary material, further inquiries can be directed to the corresponding authors.
